# Euglycemic Diabetic Ketoacidosis Precipitated by SGLT-2 Inhibitor Use, Pericarditis, and Fasting: A Case Report

**DOI:** 10.5811/cpcem.2020.4.46056

**Published:** 2020-06-15

**Authors:** Rebecca A. Mendelsohn, Anabelle N. Taveras, Benjamin A. Mazer, Lisa M. Clayton

**Affiliations:** Florida Atlantic University Charles E. Schmidt College of Medicine, Department of Emergency Medicine, Boca Raton, Florida

**Keywords:** Euglycemic DKA, SGLT-2 Inhibitor, Pericarditis, Fasting

## Abstract

**Introduction:**

Diabetic ketoacidosis (DKA) is a potentially life-threatening complication of diabetes mellitus. Less prevalent is euglycemic DKA (eDKA)—DKA with serum glucose less than 200 mg/dL; however, it is increasing in frequency with the introduction of sodium glucose cotransporter 2 (SGLT-2) inhibitors for treatment of type 2 diabetes.

**Case Report:**

We report a case of SGLT-2 inhibitor-associated eDKA presenting with concurrent acute pericarditis.

**Discussion:**

Our case suggests that the cause of eDKA can be multifactorial when decreased oral intake occurs in the setting of an acute cause of physiologic stress.

**Conclusion:**

Prompt recognition of eDKA in the emergency department may allow earlier diagnosis and treatment directed at one or more of its underlying causes.

## INTRODUCTION

Since their advent within the past decade, sodium glucose cotransporter 2 (SGLT-2) inhibitors, such as canagliflozin, empagliflozin, and dapagliflozin have gained considerable traction as a result of their highly favorable therapeutic indications. These medications have revolutionized diabetic treatment protocols by lowering patients’ blood glucose, blood pressure, and uric acid, while also promoting weight loss, and improving patients’ cardio-renal outcomes in patients with type 2 diabetes.^1^,^2^ The most dangerous complication of SGLT-2 inhibitors is possible precipitation of euglycemic DKA (eDKA), a state of DKA in which the serum glucose level is grossly normal (less than 200 mg/dL). While providers are becoming more and more aware of this clinical entity as of recently, many of these cases have gone undiagnosed or misdiagnosed.

In the past few years, reported cases of eDKA have risen sharply, in part due to increasing physician familiarity with the diagnosis as well as the increased number of SGLT-2 inhibitors being prescribed. Other independent factors that predispose patients to eDKA are similar to risk factors for classic DKA and include decreased oral intake, insulin reduction/cessation, infections, hepatic, cardiac, or renal insults, pancreatitis, and alcohol intake.^3^ The presence of multiple risk factors for eDKA should raise the index of suspicion for this diagnosis in the emergency department (ED). eDKA is a diagnostic challenge because normal blood glucose levels may lead to a false reassurance of a patient’s clinical stability, an inappropriately low triage priority, and even a delay in the initiation of critical treatment.^5^ Prompt recognition and workup of clinically significant acidosis with quantitative serum ketone measurement is crucial to the diagnosis and management of eDKA in the ED. We believe this to be the first reported case of pericarditis-associated eDKA in the setting of SGLT-2 inhibitor use.

## CASE REPORT

A 39-year-old male with a history of hyperlipidemia and non-insulin-dependent type 2 diabetes mellitus managed with metformin and empagliflozin presented to our ED complaining of three days of substernal chest pain. He reported that the pain was constant, worsening on inspiration and while leaning forward, and associated with palpitations. The patient also disclosed that for the preceding 10 days he had been maintaining a strict daytime fast for the religious holiday of Ramadan. He denied radiation of his pain, infectious symptoms, neurological symptoms, shortness of breath, abdominal pain, or dysuria. He also denied any history of connective tissue disorder. He had no recent surgical history. His family history was significant for a father with type 2 diabetes, chronic kidney disease, and early coronary artery disease. The patient denied use of alcohol, tobacco, or illicit drugs of any kind. His only recent travel was a four-hour flight two weeks prior.

On initial evaluation, the patient was afebrile, tachycardic, and hypertensive (97.8^o^ Fahrenheit, 116 beats per minute, 148/96 millimeters of mercury (mm Hg), 15 breaths per minute, oxygen saturation of 100% on 2 liters nasal cannula). His cardiac examination revealed tachycardia with normal first and second heart sounds, no murmurs, rubs, or gallops. He had normal capillary refill in his extremities and no peripheral edema. The rest of his physical examination was otherwise benign. Initial electrocardiogram revealed sinus tachycardia, significant ST-segment elevation in the precordial leads, minimal ST-segment elevation in the limb leads, ST depression in aVR, and widespread PR-segment depression. There was no evidence of ectopy, and the axis and intervals were otherwise normal. Additional workup included basic laboratory studies, cardiac enzymes, inflammatory markers, chest radiograph, and computed tomography angiography (CTA) of the chest.

Initial workup revealed leukocytosis of 13.1 × 10^9^ per liter (/L) (5.0–10.0 × 10^9^/L), negative troponin, and elevated inflammatory markers with C-reactive protein of 30.43 milligrams per deciliter (mg/dL) (0–0.74 mg/dL) and erythrocyte sedimentation rate of 75 millimeters per hour (mm/h) (0–20 mm/h). CTA of the chest revealed tiny pericardial effusion with some inflammatory changes suspicious for pericarditis and no evidence of pulmonary emboli. These findings strongly supported the diagnosis of pericarditis, and he was given a dose of intravenous toradol with subsequent improvement in his chest pain. Critically, he also had a high anion-gap acidosis with bicarbonate of 12 millimoles per liter (mmol/L) (22–32 mmol/L), anion gap of 22 milliequavalents per liter (mEq/L) (5–15 mEq/L), serum glucose of 158 mg/dL (74–118 mg/dL) and an arterial blood gas showing a pH of 7.22 (7.35–7.45), partial pressure of carbon dioxide 20.5 mmHg (35–45 mmHg), partial pressure of oxygen 115.3 mmHg (75–100 mmHg), and bicarbonate of 8.3 mmol/L (22–27 mmol/L). His lactate was normal at 1.3 mmol/L (.5–2.2 mmol/L). Cognizant of recent reports of eDKA associated with SGLT-2 inhibitors such as empagliflozin, we sent a serum beta-hydroxybutyrate level which returned at 116.1 mg/dl (0.21–2.8 mg/dL), confirming our suspicion for eDKA. His empagliflozin was stopped immediately, and he was admitted to the intensive care unit on intravenous (IV) drips of insulin and dextrose-containing maintenance fluid.

**CPC-EM Capsule**

What do we already know about this clinical entity?

Euglycemic diabetic ketoacidosis (eDKA) is a form of ketoacidosis most closely associated with use of sodium glucose cotransporter 2 (SGLT-2) inhibitors.

What makes this presentation of disease reportable?

This patient presented with eDKA in the setting of SGLT-2 inhibitor use, daytime fasting, and acute pericarditis.

What is the major learning point?

Patients taking SGLT-2 inhibitors can develop eDKA in the setting of even mildly decreased oral intake if another physiologic stressor is present.

How might this improve emergency medicine practice?

Prompt recognition of eDKA in the emergency department may allow earlier diagnosis and treatment directed at one or more of its underlying causes.

While being treated for eDKA, the patient’s pericarditis was treated with oral colchicine. Over the next two days, his acidosis improved and his anion gap closed. However, he developed a large pericardial effusion, which required emergency pericardiocentesis with placement of a pigtail pericardial drain. Serum polymerase chain reaction was positive for multiple strains of coxsackievirus A and B. The patient was discharged home on hospital day five with his medication changed to metformin and glipizide.

## DISCUSSION

There is abundant evidence that SGLT-2 inhibitors lower patients’ overall risk of myocardial infarction and stroke.^6^,^7^ Unlike many other diabetic medications, they improve morbidity and mortality without posing a significant risk of hypoglycemia.^8^ The global prevalence of these medications will surely increase in the coming years, emphasizing the importance of widespread emergency physician (EP) awareness of eDKA recognition and management.

A number of expert-written position papers argue that the ample benefits of SGLT-2 inhibitors outweigh the nominal risk of eDKA.^5^ However, in 2015 the US Food and Drug Administration released drug safety warnings about the risk of eDKA with the use of SGLT-2 inhibitors.^9^ Factors known to cause SGLT-2 inhibitor-associated eDKA include decreased oral intake, increased alcohol consumption, surgery, illness, glycogen storage disorders, and pregnancy.^10^ Specifically, decreased oral intake is the most frequently cited precipitant.^5^ However, our patient had managed to avoid any serious complications for a number of years despite taking an SGLT-2 inhibitor while fasting. It was not until he presented with acute pericarditis in the setting of fasting that he developed eDKA. This highlights the observation that similar to classic DKA, any physiologic stressor can serve as a precipitating factor for eDKA.^5^

SGLT-2 inhibitors block the reabsorption of 30–50% of filtered glucose in urine by competitive inhibition of the proximal convoluted tubule and thereby increasing urinary glucose excretion.^11^ The hypoglycemic effect of this carbohydrate deficit renders a metabolic shift from glucose utilization to lipid utilization. The lower blood glucose causes a decrease in circulating insulin and an increase in glucagon, hence a downward shift in the insulin:glucagon ratio. This leads to relative hyperglucagonemia, thereby promoting lipolysis fatty acid metabolism and ketogenesis. Decreased urinary excretion of ketones also contributes to ketonemia. Diet restriction works in an analogous way to stimulate glucagon secretion leading to reduced glycogen reserves and increased free fatty acid metabolism and ketogenesis.^12^ Diabetic patients taking SGLT-2 inhibitors already have decreased glycogen reserves. Any factor that further exacerbates this metabolic state can serve as a catalyst for eDKA.

Physiologic stressors such as illness/surgery increase the counter-regulatory hormones adrenaline and cortisol, thereby promoting increased insulin resistance and protein catabolism.^13^ In addition, physiologic stress causes increased stimulation of α1- and β-adrenergic receptors on pancreatic α-cells, promoting glucagon secretion. The reduced insulin:glucagon ratio promotes lipolysis, hepatic fatty acid oxidation, and ketogenesis. In fact, hyperglucagonemia is widely considered a surrogate marker for physiological stress. In a mechanism similar to fasting, acute pericarditis, or any physiologic stressor for that matter, can potentially bring about clinically significant ketoacidosis.

The prognosis of ketoacidosis depends largely on how expediently it is recognized and treated. Increased use of SGLT-2 inhibitors has directly correlated with the increased incidence of eDKA. Therefore, the workup of diabetic patients presenting with nausea, vomiting, abdominal pain, dyspnea, lethargy, and unexplained acidosis should include quantitative serum ketone measurement, even in the setting of normal glucose levels. Urine ketone assessment is insufficient to screen for the diagnosis because it only measures acetoacetate when the predominant ketone body in eDKA is B-hydroxybutyrate.^14^ After immediate cessation of SGLT-2 inhibitors, the treatment for eDKA is virtually identical to that of classic DKA, with the exception that dextrose-containing IV fluids must be initiated at the same time as insulin to prevent hypoglycemia.^15^

## CONCLUSION

Prescriptions for oral SGLT-2 inhibitors have increased in recent years as a result of their favorable therapeutic profile. However, cases involving potentially lethal eDKA have increased throughout the United States. Providers need to be vigilant about prescribing SGLT-2 inhibitors to those who are at risk of physiologic stress such as fasting, dehydration, extreme temperature exposure, strenuous exercise, illness, surgery, or infection. Perhaps more importantly, EPs need to maintain a high index of suspicion when diabetics taking SGLT-2 inhibitors present with traditional DKA symptoms. Our case illustrates that once a diagnosis of eDKA is made, EPs still need to carefully assess for concurrent physiologic stressors that could affect overall morbidity and mortality.
